# Effect of crystal plane orientation on the friction-induced nanofabrication on monocrystalline silicon

**DOI:** 10.1186/1556-276X-8-137

**Published:** 2013-03-25

**Authors:** Bingjun Yu, Linmao Qian

**Affiliations:** 1 Tribology Research Institute, National Traction Power Laboratory, Southwest Jiaotong University, Chengdu, 610031, People's Republic of China

**Keywords:** Friction-induced nanofabrication, Silicon crystal plane, Atomic force microscope

## Abstract

Although monocrystalline silicon reveals strong anisotropic properties on various crystal planes, the friction-induced nanofabrication can be successfully realized on Si(100), Si(110), and Si(111) surfaces. Under the same loading condition, the friction-induced hillock produced on Si(100) surface is the highest, while that produced on Si(111) surface is the lowest. The formation mechanism of hillocks on various silicon crystal planes can be ascribed to the structural deformation of crystal matrix during nanoscratching. The silicon crystal plane with lower elastic modulus can lead to larger pressed volume during sliding, facilitating more deformation in silicon matrix and higher hillock. Meanwhile, the structures of Si-Si bonds on various silicon crystal planes show a strong effect on the hillock formation. High density of dangling bonds can cause much instability of silicon surface during tip disturbing, which results in the formation of more amorphous silicon and high hillock during the friction process. The results will shed new light on nanofabrication of monocrystalline silicon.

## Background

Because of its excellent mechanical and electronic property, monocrystalline silicon has been widely used as the structural material in micro/nanoelectromechanical systems (MEMS/NEMS) [1,2]. In the past years, photolithography served as a prevailing approach to fabricate various functional micro/nanostructures on silicon surface [3,4]. However, the continuous scaling down of device size has eventually brought the photolithography to its limit in realizing high resolution [5,6]. Development of new nanofabrication methods is always a significant issue of concern. Recently, the friction-induced nanofabrication was proposed to produce protrusive nanostructures on Si(100) surface by scanning a diamond tip on a target sample without any post-etching [7]. Besides silicon, this method can also enable the fabrication on electrical insulators, such as quartz and glass. As a straightforward and maskless method, the friction-induced nanofabrication points out a new route in fabricating nanostructures on demand.

It is well known that monocrystalline silicon has three typical crystal planes, i.e., (100), (110), and (111). As a typically anisotropic material, monocrystalline silicon presents different elastic modulus on various crystal planes, namely 130 GPa on Si(100), 169 GPa on Si(110), and 188 GPa on Si(111), respectively [8]. Experimental results showed that the cutting process and friction behavior of silicon were influenced by the crystal anisotropy [9,10]. Based on pin-on-disk tests, the average friction coefficient measured on Si(100) wafer was about 80% higher than that on Si(110) and Si(111) wafers [10]. Moreover, because of the difference in the density of dangling bonds and structure of back bonds, the etching rate of Si(100) or Si(110) was two orders of magnitude faster than that of Si(111) in alkaline solution [11,12]. These anisotropic properties of monocrystalline silicon may induce the different nanofabrication behavior on silicon surfaces with various crystal planes. Therefore, even though the friction-induced nanofabrication enables producing protrusive nanostructures on Si(100) surface, it remains unknown whether the same nanofabrication method can be realized on other silicon crystal planes.

In the present study, the effect of crystal plane orientation on the friction-induced nanofabrication on monocrystalline silicon was investigated. To verify whether the friction-induced fabrication can be realized on various silicon crystal planes, scratch tests at a linearly increasing load were performed on Si(100), Si(110), and Si(111) surfaces, respectively. The effect of crystal plane orientation on the formation of friction-induced hillocks was further detected by scanning three silicon crystal planes under a constant normal load. Finally, the formation mechanism of the hillock on various silicon crystal planes was discussed based on their mechanical performance and bond structure.

## Methods

### Materials

Si(100), Si(110), and Si(111) wafers were purchased from MCL Electronic Materials Ltd., Luoyang, China. The surface root-mean-square roughness of the wafers was measured as less than 0.2 nm over a square of 2 × 2 μm^2^ by an atomic force microscope (AFM; SPI3800N, Seiko Instruments Inc., Tokyo, Japan). The mechanical properties of the wafers were detected by a triboindenter (TI750, Hysitron Inc., MN, USA). During the indentation tests, a spherical diamond indenter with the nominal curvature radius *R* = 1 μm was used, and the maximum indentation depth was set to 20 nm.

### Nanofabrication tests

To investigate whether the friction-induced nanofabrication can be realized on silicon surfaces with various crystal planes, the scratches were performed on Si(100), Si(110), and Si(111) surfaces by a nanoscratching tester (NST; CSM Instruments SA, Peseux, Switzerland) in air. A diamond tip with *R* = 2 μm was employed, and the scratching distance was 200 μm. Since the minimum load applied by the tester was 0.3 mN and surface grooves can be produced on silicon wafers at 6.0 mN, the scratching test was performed under linear loading from 0.3 to 6.0 mN. Before the fabrication tests, the silicon wafers were ultrasonically cleaned with acetone, ethanol, and deionized water in turn to remove surface contamination.

To study the effect of crystal plane orientation on the hillock formation on silicon, the fabrication was performed on three silicon samples by AFM with a vacuum chamber under a constant load (*F*_n_) of 50 μN both in air and in vacuum (<5.0 × 10^−6^ Torr). A diamond tip (Micro Star Technologies, TX, USA) with *R* = 500 nm was used. The normal spring constant (*k*) of the cantilever of the AFM diamond tip was calibrated as 194 N/m through a calibration cantilever (CLFC-NOBO, Veeco Instruments Inc., NY, USA) [13]. The line-shaped hillocks were produced at the sliding velocity of 40 μm/s. The number of scratch cycles (*N*) was 100 or 200. To study the effect of pressed volume on the hillock formation, a sharp diamond tip (*R* = 250 nm) was employed to perform the fabrication test on Si(100) surface in air. The topography of the scratches produced by the NST and the hillocks by the AFM was observed using the silicon nitride tips (MLCT, Veeco Instruments Inc.) with *R* = 20 nm and *k* = 0.1 N/m. During the entire experimental process, the temperature was set to 25 ± 2°C, and the relative humidity was between 50% and 55%.

## Results

### Realization of friction-induced nanofabrication on various silicon crystal planes

**Figure 1:**
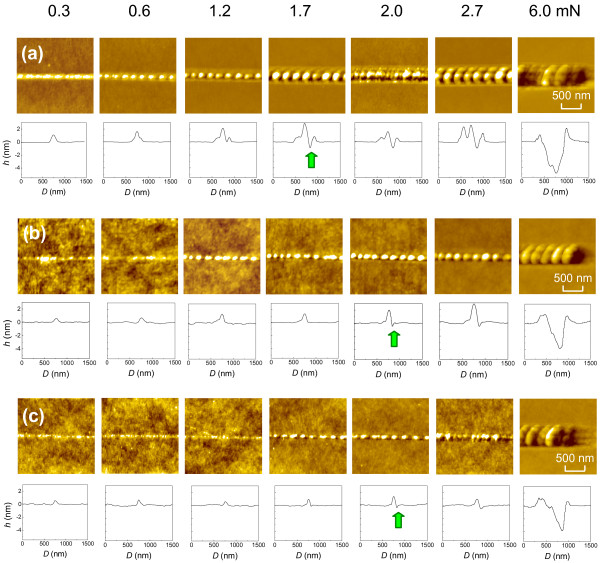
**Evolution of the scratches on (a) Si(100), (b) Si(110), and (c) Si(111) surfaces.** The scratches were produced at a linearly increasing load from 0.3 to 6.0 mN. Each AFM image (2 × 2 μm^2^) was taken from the appointed segment of the same scratch on silicon with a given crystal plane. The arrows on the cross-sectional profiles indicate the appearance of the groove.

When a silicon surface was scratched by a sharp diamond tip at relatively high normal loads, the groove was usually produced along the scratching trace [14]. To verify whether the protrusive hillock can be generated on the silicon surfaces with various crystal planes, scratching tests were conducted on Si(100), Si(110), and Si(111) surfaces under linear loading from 0.3 to 6.0 mN, respectively. As shown in Figure 1, under a relatively low normal load, friction-induced hillocks can be generated on these silicon surfaces regardless of their anisotropic properties. With the increase in the applied normal load, all the scratches on the three silicon crystal planes change gradually from hillock to groove. The result is consistent with the transition of hillock to groove observed on Si(100) surface by repeated scratching [7]. From the profiles in Figure 1, the normal load (*F*_n_) that lead to the appearance of groove is approximately 1.7 mN on Si(100) surface and 2.0 mN on the other two crystal planes (indicated by arrows). Based on the Hertzian contact model [15], the corresponding maximum contact pressure (*P*_0_) was estimated as 10.9 GPa for Si(100), 13.4 GPa for Si(110), and 14.2 GPa for Si(111), respectively. Since the hardness of Si(100), Si(110), and Si(111) was measured as 11.3, 13.0, and 13.2 GPa with the triboindenter, the calculated critical pressure is very close to the hardness of monocrystalline silicon with different crystal planes [8,16]. With the increase in *F*_n_, although the value of *P*_0_ attains to that of the hardness, the average pressure on the contact area may be still lower than that on the hardness. Hence, the scratch with both hillock and groove will be produced, and the hillock will become larger as the load increased. With the further increase in the load, groove formation will be dominant, and hillock will disappear because of the severe plastic deformation. Therefore, when the contact pressure is less than the hardness of the monocrystalline silicon, the friction-induced hillock can be created on silicon surfaces with various crystal planes.

### Comparison of hillock formation under the constant load

**Figure 2:**
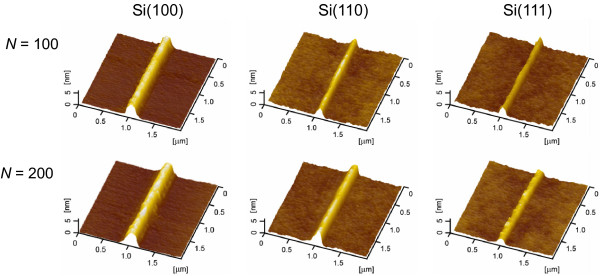
**AFM images of the friction-induced hillocks on Si(100), Si(110), and Si(111) surfaces produced in air.** The *F*_n_ is 50 μN, and the *N* is 100 and 200.

**Figure 3:**
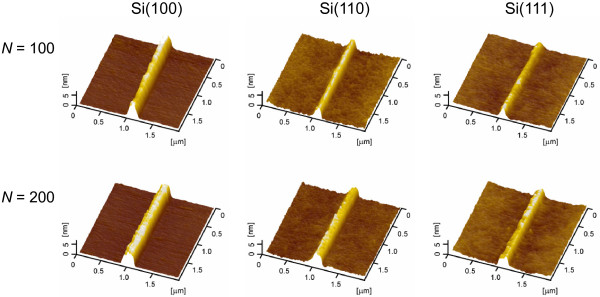
**AFM images of the friction-induced hillocks on Si(100), Si(110), and Si(111) surfaces produced in vacuum.** The *F*_n_ is 50 μN, and the *N* is 100 and 200.

Although the friction-induced fabrication can be realized on silicon surfaces with various crystal planes, the friction-induced hillocks on various silicon crystal planes are a little different, as shown in Figure 1. To accurately compare the hillock formation on various silicon surfaces, the scratch tests were performed on three silicon crystal planes under the same constant load by AFM both in air and in vacuum. As shown in Figures 2 and 3, the hillocks were created on three silicon crystal planes under a constant load of 50 μN, where the contact pressure was estimated as 8.5 to 10.5 GPa. Figure 2 shows the hillocks produced in air with *N* of 100 and 200, respectively. Under the same loading condition, the hillock formation was also investigated in vacuum, as shown in Figure 3.

**Figure 4:**
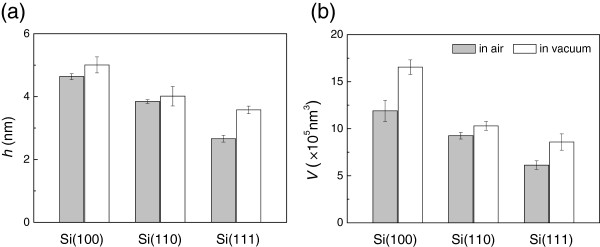
**Comparison of the (a) height (*h*) and (b) volume (*V*) of the friction-induced hillocks.** The hillocks were produced at *F*_n_ = 50 μN and *N* = 100 in air and in vacuum, respectively.

To quantitatively compare the hillock size on various silicon crystal planes, the height and volume of the hillocks were measured with the original silicon surface as the base level. As shown in Figure 4, the hillock produced on Si(100) surface was the highest, but that produced on Si(111) surface was the lowest either in air or in vacuum. For example, the hillock produced in air/vacuum at *N* = 100 on Si(111) surface is 42%/29% lower than that on Si(100) surface. The hillocks produced at *N* = 200 show the similar results. It was also noted that the hillock produced in air was a little lower than that in vacuum, which may be to some extent ascribed to the protective effect of surface oxide layer on the silicon surface [17]. Since less silicon oxide layer was observed on the hillock surface when scratched in vacuum than that in air, taller hillocks would be created in vacuum [18]. In summary, because of the anisotropic properties of silicon surfaces, the friction-induced hillocks on Si(100) surface were the highest, but those on Si(111) surface were the lowest under the same conditions. The reasons responsible to the difference will be further discussed in the next section.

## Discussions

### Effect of the mechanical property on the hillock formation

The transmission electron microscope observation indicated that the friction-induced hillock on Si(100) surface contained a thin superficial oxidation layer and a thick disturbed (amorphous and deformed) layer in the subsurface [17,18]. It was suggested that the mechanical interaction through amorphization was the key contributor to hillock formation on Si(100) surface. Although the silicon wafers with various crystal planes present different elastic modulus, all these wafers consist of Si-I phase (diamond-like structure) regardless of crystallographic orientations. During the sliding process, the transformation of Si-I to amorphous structure may occur on three silicon crystal planes, which will further induce the formation of hillock on these silicon surfaces. However, under the same loading conditions, the height of hillock on various silicon crystal planes was different as shown in Figures 2, 3 and 4. The results suggested that the crystal plane orientation of silicon had a strong impact on the friction-induced nanofabrication on the silicon surface.

Due to the anisotropic mechanical properties of monocrystalline, the tip-sample contact may be different on three silicon crystal planes during scratching. When the scratch test was performed at *F*_n_ = 50 μN, the maximum shear stress on the contact area was estimated as 2.6 GPa on Si(100), 3.1 GPa on Si(110), and 3.3 GPa on Si(111) with the Hertzian contact model, respectively [15]. Since all the shear stress was below the yield stress of silicon (approximately 7 GPa), the deformation during the scratch process on the three silicon crystal planes was assumable to be elastic according to the Tresca yield criterion [19]. However, the repeated scanning under low load may lead to the deformation of silicon matrix, i.e., the formation of amorphous layer and stacking faults, which may in turn induce the generation of hillock [17]. When scratching a diamond tip under the same loading condition, silicon crystal plane with lower elastic modulus will induce larger contact area and more pressed volume, which provides more probability for deformation of silicon matrix below the scratching tip. As shown in Table 1, since the elastic modulus of Si(100) surface is 23%/31% lower than that of Si(110)/Si(111) surface, the pressed volume on Si(100) is 36%/53% larger than that on Si(110)/Si(111) surface at *F*_n_ = 50 μN.

**Table 1:** Comparison of the contact of a diamond tip on various silicon crystal planes

**Sample**	**Si(100)**	**Si(110)**	**Si(111)**
Contact area *A* (nm^2^)	8.86 × 10^3^	7.61 × 10^3^	7.17 × 10^3^
Pressed volume *V* (nm^3^)	2.49 × 10^4^	1.83 × 10^4^	1.63 × 10^4^

The tip radius (*R*) is 500 nm, and the normal load (*F*_n_) is 50 μN.

**Figure 5:**
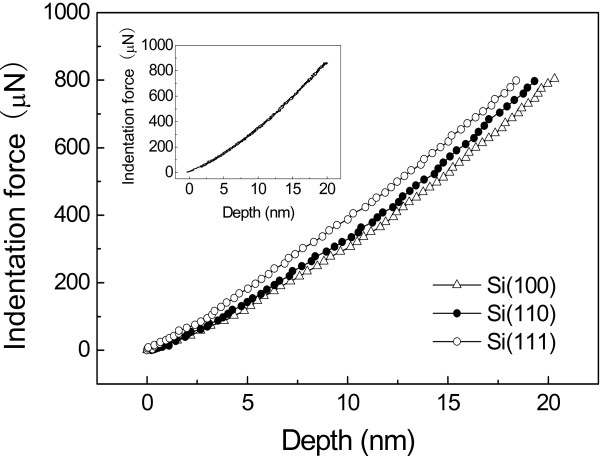
**Comparison of the indentation force-depth curves on Si(100), Si(110), and Si(111) surfaces.** Indentation force-depth curves during loading process measured by a diamond tip with *R* = 1 μm. The inset showed that the indentation force-depth curves on Si(100) surface during loading and unloading process overlapped with each other, suggesting that the deformation during indentation process was purely elastic.

Such results can be further confirmed by the indentation tests with a spheric diamond tip (*R* = 1 μm). As shown in Figure 5, since the measured loading/unloading curves were overlapped at the maximum indentation depth of 20 nm, the deformation during the indentation process was purely elastic. At the same indentation force, the indentation depth and the pressed volume on Si(100) surface were the largest, while those on Si(111) surface were the smallest. The larger pressed volume provides more probability for deformation of silicon matrix below the scratching tip. Therefore, the highest/lowest hillock was produced on Si(100)/Si(111) in the present study.

**Figure 6:**
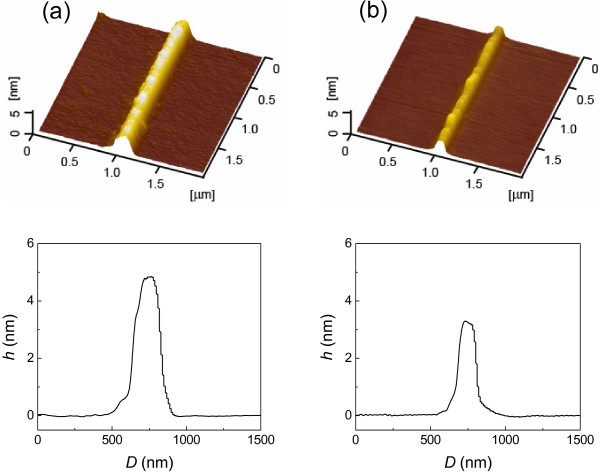
**Comparison of the hillocks produced with different diamond tips under the same contact pressure.** (**a**) *R* = 500 nm; (**b**) *R* = 250 nm. The number of scratch cycles was 100.

The effect of pressed volume on the hillock height can be further verified by the fabrication tests with different diamond tips. As shown in Figure 6, friction-induced hillocks were produced on Si(100) surface with two different diamond tips (*R*=500 and 250 nm) under the same contact pressure (8.5 GPa). The hillock produced by the blunt tip was 4.9 nm in height, while the hillock produced by the sharp tip was only 3.3 nm in height. When the pressed volume increased by 692%, the height of the produced hillock increased by 48%. Clearly, the pressed volume had a strong effect on the hillock formation. The larger pressed volume corresponds to the formation of more amorphous silicon and higher hillock.

### Effect of the surface bond structure on the hillock formation

**Figure 7:**
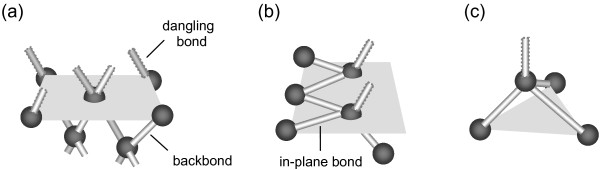
**Configuration of Si-Si covalent bonds on different planes of monocrystalline silicon.** (**a**) Si(100); (**b**) Si(110) and (**c**) Si(111). The dangling bonds were indicated by dotted lines. Some covalent bonds that inhibit on one atom are partly showed.

It was reported that various silicon crystal planes showed strong anisotropic behavior in etching by alkaline solutions [11,12,20] and in tribological tests [10,21]. Such behaviors were mainly attributed to the difference in the density of the dangling bonds as well as the backbonds on the silicon surface [12]. As shown in Figure 7, the dangling bonds inhabit on the superficial layer of a given crystal plane, and the backbonds lie in the subsurface of the plane as well as the in-plane bonds. The dangling bond is partly bonded to the silicon atom beneath and leads to a metastable surface matrix [22]. Compared with Si-Si bonds in the subsurface, the dangling bond is speculated to be easily bended and rolled during scratching. Such instability provides an effective channel on the given silicon plane for the energy input, resulting in the formation of more amorphous silicon and higher hillock [17]. Crystal plane with higher density of dangling bonds can cause much instability and can lead to higher hillock during scratching.

With two dangling bonds on each silicon atom, the (100) plane has the highest density of dangling bonds compared with the other crystal planes. Although only one dangling bond is attached to one silicon atom, the nonequilibrium in bonding state is further increased by the in-plane bonds on (110) plane [23]. Even with the similar dangling bond number per atom as the (110) plane, the atom on the (111) plane is supported by three equivalent Si-Si backbonds, which enhance the mechanical stability of the Si(111) surface [21,24]. Therefore, under the same loading condition, the highest hillock was generated on Si(100), while the lowest hillock was formed on Si(111) either in air or in vacuum. However, the disturbance from the tip was reduced because of the protective effect of the adsorbed water, oxidation layer, and contamination in air. As a result, a little lower hillock was produced on silicon in air compared to that in vacuum.

In summary, the friction-induced nanofabrication can be realized on different silicon crystal planes, with the contact pressure less than the hardness. At the same normal load, the silicon crystal plane with low elastic modulus or high density of dangling bonds can facilitate the formation of friction-induced hillock. Because of the configuration of Si-Si bonds, crystal silicon reveals different mechanical properties on various crystal planes, which eventually result in the variation of hillock formation in the present study. These findings may provide possibilities to control the hillock formation on monocrystalline silicon and help understand the subtle mechanism.

## Conclusions

Nanofabrication tests were performed contrastively on Si(100), Si(110), and Si(111) surfaces using diamond tips. The formation of friction-induced hillocks on various silicon crystal planes was observed both in air and in vacuum conditions. The effect of the crystal plane orientation on the friction-induced nanofabrication was mainly attributed to the different mechanical behaviors and bond structures of the various silicon crystal planes. The main conclusions can be summarized as below.

(1) Friction-induced nanofabrication can be realized on Si(100), Si(110), and Si(111) surfaces, respectively. The crystal plane orientation has a significant effect on the hillock formation on silicon surface. Under the same loading condition, the highest hillock was generated on Si(100), while the lowest hillock was formed on Si(111) either in air or in vacuum.

(2) The mechanical performance of silicon shows a strong effect on the hillock formation on various silicon crystal planes. The crystal plane with the lower elastic modulus can lead to larger pressed volume, which facilitates more deformation in silicon matrix and higher hillock.

(3) The structures of Si-Si bonds play a key role in the hillock formation on various silicon crystal planes. High density of dangling bonds can cause much instability, facilitating the formation of more amorphous silicon and high hillock during nanoscratching.

## Abbreviations

AFM: atomic force microscope; Fn: applied normal load; h: height; k: normal spring constant; MEMS: microelectromechanical systems; N: number of scratch cycles; NEMS: nanoelectromechanical systems; NST: nanoscratching tester; R: curvature radius.

## Competing interests

The authors declare that they have no competing interests.

## Authors’ contributions

BY finished the fabrication experiments and acquired the original data in this article. LQ has made substantial contributions to conception and design for this article. Both authors read and approved the final manuscript.
